# Photoplethysmography acceleration indices as digital biomarkers of cardiometabolic health

**DOI:** 10.3389/fdgth.2026.1910212

**Published:** 2026-08-26

**Authors:** Gianluca Diana, Corin F. Otesteanu, Francesco Scardulla, Salvatore Pasta, Leonardo D’Acquisto, Carlo Menon

**Affiliations:** 1 Department of Engineering, University of Palermo, Palermo, Italy; 2 Biomedical and Mobile Health Technology Lab, ETH Zürich, Zürich, Switzerland

**Keywords:** acceleration plethysmogram (APG), diabetes, hypertension, optical sensors, photoplethysmography (PPG), signal processing, vascular aging, wearable devices

## Abstract

Non-invasive monitoring of cardiovascular health is critical for early detection of dysfunction. Photoplethysmography (PPG) is already widely used in wearable devices to track cardiovascular signals, such as heart rate and pulse wave characteristics. The second derivative of the PPG signal, known as the accelerated plethysmogram (APG), provides indices reflecting arterial stiffness and vascular aging. Despite their potential as digital biomarkers, systematic comparisons across clinical populations are limited. In this cross-sectional study, four APG-derived indices (b/a, c/a, d/a, AGI) were analyzed in 133 participants: healthy individuals (*n* = 45), people with hypertension (*n* = 68), and people with diabetes (*n* = 20). All indices correlated significantly with chronological age within each group. Compared with healthy individuals, both pathological groups showed marked differences across indices, with large effect sizes. Direct comparison between participants classified as hypertensive and those classified as diabetic, with or without hypertension, revealed that the b/a ratio was the most discriminative parameter between these clinical groups (Cohen's |*d*| = 1.19). These findings indicate that APG indices differ significantly across health states and may represent non-invasive digital biomarkers of vascular health, supporting their integration into wearable devices for continuous cardiovascular monitoring.

## 1 Introduction

Cardiovascular diseases (CVDs) are the leading cause of death globally (1). According to data from the World Health Organization, CVDs caused approximately 19.2 million deaths in 2023, accounting for one-third of all deaths worldwide (2). Over 80% of these deaths are attributable to ischemic heart disease and stroke, and about one-third occur prematurely in individuals under the age of 70 (3). High blood pressure is the main risk factor for CVD, contributing to approximately 10 million deaths annually (4), while diabetes mellitus and obesity are metabolic risk factors that are constantly growing at an epidemiological level (5).

In this context, assessing the health of the vascular system plays a fundamental role in the prevention, early identification and stratification of cardiovascular risk (6). Arterial stiffness, in particular, has emerged as an independent marker of cardiovascular risk (7), capable of predicting events such as myocardial infarction and stroke, independently of traditional risk factors (8, 9). Arterial stiffening is a process in which the arteries undergo progressive dilation and loss of elasticity, with important hemodynamic consequences including elevated pulse pressure, increased left ventricular afterload, and impaired coronary perfusion (10). This phenomenon reflects both the cumulative vascular damage caused by vascular ageing (11), which does not necessarily coincide with chronological age (12), and the deleterious effects of pathological conditions such as hypertension, diabetes and atherosclerosis on the arterial wall (13, 14).

Currently, carotid-femoral pulse wave velocity (cfPWV) is considered the gold standard for non-invasive assessment of arterial stiffness (15, 16). However, this method requires dedicated equipment and specialised personnel, limiting its applicability in large-scale clinical and population screening. Therefore, in recent years, there has been growing interest in alternative methods that are simpler, low cost, non-invasive and easily implemented in wearable devices for outpatient monitoring of cardiovascular health, among which photoplethysmography has emerged as a prominent approach (17, 18).

Photoplethysmography (PPG) is a non-invasive optical method that measures blood volume changes in a microvascular bed of the skin by detecting changes in light absorption (19). This method is based on the emission of light (typically red, infrared or green) into the tissue and the measurement of transmitted or reflected light, which varies according to the pulsatile blood flow in the vascular bed (20). PPG sensors are now widely used and integrated into consumer devices such as pulse oximeters (21), smartwatches (22), smartbands (23, 24), and smart rings (25), making them particularly attractive for continuous cardiovascular monitoring applications. In addition to traditional applications such as heart rate monitoring (26) and oxygen saturation estimation (27), PPG-based techniques have been increasingly explored for a wide range of cardiovascular assessments, including non-invasive blood pressure estimation (28–30), atrial fibrillation detection (31), and autonomic nervous system function assessment (32). Furthermore, the morphology of the PPG signal, whose quality and reliability are influenced by physiological and technical factors such as the contact pressure between the sensor and the skin surface (33), skin temperature variations (34), as well as the subject's level of physical activity (35), contains clinically relevant information related to left ventricular ejection (36), arterial compliance (37), vascular stiffness (38, 39), and arterial ageing (40). Recent advanced studies have also demonstrated the potential of PPG signals combined with machine learning algorithms for monitoring transcatheter heart valve function, enabling non-invasive detection of valve prosthesis deterioration (41). Furthermore, *in vitro* investigations have demonstrated that PPG sensors can effectively assess pressure gradients across bioprosthetic valves by analysing pulse transit time and pulse wave velocity, opening the way for cost-effective monitoring systems in clinical practice (42).

In recent years, an approach that has gained increasing relevance in the scientific community is the analysis of the second derivative of the photoplethysmographic signal (SDPPG), commonly referred to as acceleration plethysmogram (APG) (43). Following the recommendations of (44), the acronym APG will be used exclusively in this study. Introduced by Takazawa et al. in 1998 (45), this technique provides a mathematical transformation of the PPG signal that highlights inflection points and rapid waveform changes, facilitating the identification of systolic and diastolic components and enabling more accurate assessment of vascular status (46–48).

The APG waveform presents five characteristic waves, named a, b, c, d and e: wave a represents the early positive systolic wave, related to the initial acceleration of blood flow during ventricular ejection; wave b corresponds to the early negative systolic wave, reflecting the deceleration phase following the peak of ejection; wave c is the late systolic re-increment wave, associated with arterial compliance and wave reflection; wave d represents the late systolic re-decrement wave, related to the timing and magnitude of the reflected waves; finally, wave e corresponds to the early positive diastolic wave, associated with the dicrotic notch and closure of the aortic valve. By combining the amplitudes of these characteristic waves, various quantitative indices can be obtained for assessing vascular status (28, 49).

In recent years, analysis of the second derivative of the photoplethysmogram has been used in an increasing number of clinical contexts, exploiting the morphological information contained in the APG signal. In addition to applications in non-invasive blood pressure estimation (50, 51), ophthalmology (52, 53) and acute cardiovascular event detection (54), APG has shown significant potential in the stratification of hypertensive patients, where alterations in arterial compliance are reflected in characteristic changes in APG indices (55). Similarly, correlations between APG indices and blood glucose levels have been documented, with implications for the development of non-invasive systems for screening and monitoring diabetes mellitus (56, 57).

Most existing studies have examined individual pathological conditions or homogeneous populations, providing important contributions to understanding the behaviour of APG indices in specific clinical contexts. However, a direct and systematic comparison between heterogeneous clinical groups, characterised by different cardiovascular risk profiles, could offer a complementary perspective, allowing us to assess the sensitivity of these indices in reflecting the different degrees of vascular impairment associated with conditions such as arterial hypertension and diabetes mellitus.

In this context, the present study analyses the indices derived from plethysmogram acceleration in a heterogeneous population of subjects stratified into three clinical groups: healthy subjects, hypertensive subjects, and diabetic subjects. The main objectives are to evaluate the discriminatory power of these indices across groups, their correlation with chronological age, and to explore the potential of APG indices as biomarkers for monitoring cardiometabolic health, with a view toward future applications in wearable non-invasive vascular monitoring.

## 2 Methods

### 2.1 Database and study population

This study used the publicly available PPG-BP (Photoplethysmography-Blood Pressure) database, a dataset developed at Guilin People's Hospital, China, by Liang et al. (58). The database includes high-quality photoplethysmographic recordings associated with biometric data and clinical information, specifically designed for non-invasive cardiovascular research.

The dataset includes 657 PPG signal segments acquired from 219 adult subjects, ranging in age from 21 to 86 years (57.2 ± 15.9 years). The gender distribution is balanced, with 48% of participants being male. The study population includes subjects with different clinical conditions: healthy subjects (normotensive), subjects with hypertension (classified as pre-hypertension, stage I and stage II according to international guidelines), diabetic subjects and subjects with cerebrovascular diseases (cerebral infarction, cerebrovascular insufficiency). The original study was approved by the ethics committee of Guilin People's Hospital and Guilin University of Electronic Technology.

PPG signals were acquired using a customised portable hardware platform consisting of a PPG probe (model SEP9AF-2, SMPLUS Company, Korea) and a microcontroller (MSP430FG4618, Texas Instruments, USA) for data acquisition and Bluetooth transmission. The PPG signals were acquired from the fingertips of subjects at rest using an infrared wavelength LED (*λ* = 905 nm) at a sampling frequency of 1 kHz and with a 12-bit ADC.

The experimental protocol included a 10-minute acclimatisation period, during which the subject remained seated in a comfortable position with their upper limbs resting on a flat surface. Subsequently, within a 3-minute time window, the PPG signal and blood pressure were simultaneously acquired using an Omron HEM-7201 electronic sphygmomanometer (Omron Company, Kyoto, Japan). The total duration of the experiment was approximately 15 min per participant.

To the best of our knowledge, this is the only publicly available PPG resource that combines the acquisition characteristics described above with physician-assigned diagnoses for normotensive, hypertensive and diabetic participants, collected in resting conditions within a single acquisition protocol. Publicly available PPG datasets with physician-assigned hypertension diagnoses collected in resting conditions do not exist outside of this resource. Similarly, while diabetes-related PPG datasets exist for prediabetic and Type 1 diabetic populations, these were collected under free-living conditions using consumer wearables and lack a matched healthy or hypertensive comparison group; to our knowledge, no such dataset exists for Type 2 diabetes. Neither alternative permits the three-group resting-state comparison performed here.

### 2.2 Study design

For the purposes of this study, subjects in the PPG-BP database were classified into three main clinical groups (Table 1):

1. Healthy subjects: normotensive individuals with no diagnosis of diabetes mellitus, arterial hypertension or documented cerebrovascular disease;
2. Hypertensive patients: subjects diagnosed with arterial hypertension (pre-hypertension, stage I or stage II) in the absence of diabetes mellitus;
3. Diabetic patients: subjects diagnosed with diabetes mellitus, regardless of the concomitant presence of arterial hypertension.

**Table 1:** Demographic and clinical characteristics of study participants.

Category	Variable	Healthy	Hypertensive	Diabetic	Total
Demographics	Age range (year)***	21–85	25–86	38–84	21–86
	Mean and std age (year)	47.8 ± 17.2	63.0 ± 13.2	60.0 ± 12.8	57.4 ± 16.1
	Male	17 (37.8%)	27 (39.7%)	10 (50.0%)	54 (40.6%)
	Female	28 (62.2%)	41 (60.3%)	10 (50.0%)	79 (59.4%)
Clinical parameters	Height (cm)	160.1 ± 8.2	160.1 ± 8.3	161.3 ± 9.2	160.3 ± 8.3
	Weight (kg)*	57.5 ± 10.5	60.9 ± 11.9	65.8 ± 12.3	60.5 ± 11.7
	BMI (kg/m^2^)*	22.4 ± 3.9	23.7 ± 4.0	25.2 ± 3.9	23.5 ± 4.0
	SBP (mmHg)***	106.7 ± 9.0	141.4 ± 14.5	131.7 ± 14.0	128.2 ± 20.3
	DBP (mmHg)***	64.0 ± 6.3	76.4 ± 11.1	74.7 ± 10.9	72.0 ± 11.2
	HR (bpm)	70.3 ± 8.9	70.1 ± 8.9	66.8 ± 8.6	69.7 ± 8.9
BP Classification	Normotensive, *n* (%)	45 (100.0%)	-	3 (15.0%)	48 (36.2%)
	Pre-hypertension, *n* (%)	–	37 (54.4%)	12 (60.0%)	49 (36.8%)
	Stage 1, *n* (%)	–	22 (32.4%)	4 (20.0%)	26 (19.5%)
	Stage 2, *n* (%)	–	9 (13.2%)	1 (5.0%)	10 (7.5%)

* *p* < 0.05.

*** *p* < 0.001.

The quality of each PPG signal segment was assessed using the Skewness Signal Quality Index $\left(S_{SQI}\right)$, defined as:

$$S_{SQI}=\frac{1}{N}\sum_{i=1}^{N}{\left[\frac{x_{i}-\hat{\mu_{x}}}{\sigma }\right]}^{3}$$

where *N* is the sample number of the PPG signal, ${\hat{\mu }}_{x}$ and *σ* are respectively the empirical estimates of the mean and standard deviation of the samples $x_{i}$ (59). Comparative studies have shown that $S_{SQI}$ is the optimal method for assessing PPG signal quality compared to other indices, such as perfusion index $\left(P_{SQI}\right)$, Kurtosis $\left(K_{SQI}\right)$, Entropy $\left(E_{SQI}\right)$ or signal-to-noise ratio $\left(N_{SQI}\right)$ (60). The $S_{SQI}$ allows signals of excellent, acceptable or unsuitable quality to be distinguished based on the morphological characteristics of the waveform (61).

In this study, only PPG segments with $S_{SQI}\geq 0.41$ were included in the analysis, in accordance with the threshold adopted by Abdullah et al. (62) for the selection of segments of adequate quality for the identification of fiducial points. This threshold ensures the presence of complete waveforms with clearly identifiable systolic and diastolic periods, allowing optimal detection of APG fiducial points.

The study included 133 participants, for a total of 399 available PPG segments. Of these, 35 segments (8.8%) were excluded for $S_{SQI}$ values below the threshold value, resulting in 364 waveforms analysed.

In all retained segments, the five fiducial points were successfully identified, and all four APG indices were computed for every subject in each of the three clinical groups.

The 133 subjects were stratified into three clinical groups: 45 healthy subjects (33.8%), 68 hypertensive subjects (51.1%) and 20 diabetic subjects (15.0%). The demographic and clinical characteristics of the study population are summarised in Table 1. The mean age of the total population was 57.4 ± 16.1 years, ranging from 21 to 86 years. Healthy subjects were significantly younger (47.8 ± 17.2 years) than hypertensive subjects (63.0 ± 13.2 years) and diabetic subjects (60.0 ± 12.8 years). The gender distribution showed a slight female prevalence in the total sample (59.4% females), with similar proportions among the clinical groups. Body mass index was lower in healthy subjects (22.4 ± 3.9 kg/m^2^) and higher in diabetic subjects (25.2 ± 3.9 kg/m^2^). Systolic blood pressure was significantly lower in healthy subjects (106.7 ± 9.0 mmHg) than in hypertensive subjects (141.4 ± 14.5 mmHg) and diabetic subjects (131.7 ± 14.0 mmHg). All these data are shown in Table 1.

### 2.3 Signal processing

**Figure 1:**
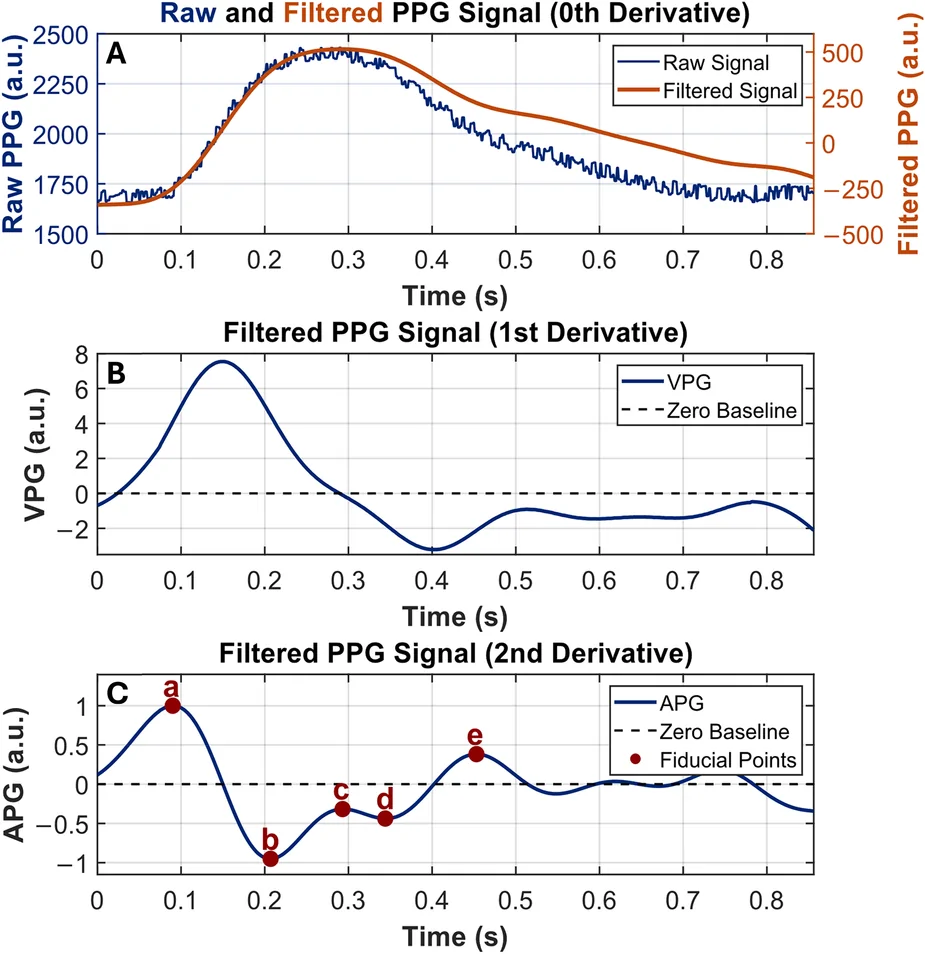
PPG signal processing and identification of APG fiducial points. **(A)** Raw (blue) and filtered (orange) PPG signals. **(B)** First derivative (VPG). **(C)** Second derivative (APG) with the five fiducial points (a, b, c, d, e).

The raw PPG signals were processed using a custom algorithm, developed *ad hoc* and implemented in MATLAB (v.2022b, The MathWorks, Inc., Natick, MA, USA), for the automated application of filtering, derivative calculation and fiducial point identification (Figure 1). The selection of processing parameters was guided by the evidence available in the literature regarding the optimisation of signal quality and the preservation of the morphological characteristics of the PPG waveform, with particular attention to the correct identification of the fiducial points of the second derivative (63–65).

The raw PPG signal was first subjected to bandpass filtering using a fourth-order Chebyshev type II filter, with a passband of 0.5–8 Hz and attenuation in the stopband of 20 dB (Figure 1A) (66). The choice of a type II Chebyshev filter was motivated by its optimal properties for processing PPG signals, as it has a sharper transition zone while maintaining a flat passband, a feature that preserves the morphological components of the original signal while minimising distortion and is therefore particularly effective in highlighting the dicrotic notch, a fundamental element for the analysis of arterial compliance and APG fiducial points (67, 68). Subsequently, the filtered signals were normalised in the amplitude range between 0 and 1 to allow inter-subject comparisons. The VPG was calculated by applying a Savitzky-Golay filter with first-order derivation, third-order polynomial and a 150 ms window to the filtered PPG signal (Figure 1B). The APG was obtained by reapplying the Savitzky-Golay filter with first-order derivation to the VPG signal (Figure 1C). The use of the Savitzky-Golay filter for calculating derivatives allows simultaneous differentiation and smoothing of the signal in a single step, intrinsically attenuating the amplification of high-frequency noise introduced by the subsequent derivation, without the need for additional filtering (49). The resulting APG signal was normalised to the maximum absolute value, resulting in an amplitude in the range between −1 and 1. This normalisation allows direct comparison of the relative amplitudes of the fiducial points between subjects belonging to different clinical groups, ensuring signal standardisation regardless of inter-individual variations in absolute amplitude.

The identification of fiducial points in the APG signal was performed using an algorithm based on the analysis of the morphological characteristics of the cardiac cycle waveform. Wave a was identified as the global maximum of the APG signal within each cardiac cycle. Wave b was identified as the global minimum of the APG signal. The c and d waves were identified, respectively, as the first local positive maximum and the first local minimum following the b wave as proposed by Abdullah et al. (67). Finally, wave e was identified as the second positive local maximum following wave b (69).

The quantitative indices derived from the APG were calculated based on the amplitude of the five fiducial points identified, according to the definitions introduced by Takazawa et al. (45). The ratios b/a, c/a and d/a were calculated by normalizing the amplitude of each wave relative to wave a. The Aging Index (AGI), calculated as (b−c−d−e)/a, integrates the information derived from all components of the APG wave into a single composite parameter.

### 2.4 Statistical analysis

To ensure statistical independence, repeated measurements were not treated as separate observations. Instead, for each subject, the average value of each APG index (b/a, c/a, d/a, and AGI) was calculated across all available repeated measurements, and all subsequent analyses were performed using these subject-level values (one observation per subject). For each parameter and for each clinical group, descriptive statistics (mean, standard deviation, and median) were calculated. The relationship between the APG indices and chronological age was assessed using Pearson's correlation coefficient (*r*) with the corresponding *p*-values, stratified by clinical group.

To compare the APG indices among the three clinical groups, a pipeline of formal statistical tests was applied. First, the assumptions required for the use of parametric tests were checked: normality of the distribution of each APG index within each group was assessed using the Lilliefors test, while homoscedasticity (homogeneity of variances) between groups was evaluated using Levene's test in the version based on the median. When both assumptions were met, ANOVA test was performed; otherwise, the non-parametric Kruskal–Wallis test was used as an alternative.

When the omnibus test indicated a statistically significant difference (*p* < 0.05), *post-hoc* pairwise comparisons were conducted: the Tukey HSD test following ANOVA, or the Dunn test with Bonferroni correction following the Kruskal–Wallis test. This approach allows for control of the overall family-wise error rate in multiple comparisons. For each pairwise comparison, Cohen's *d* was calculated as a measure of effect size, using the pooled standard deviation. Effect sizes were interpreted according to the conventional thresholds proposed by Cohen (70, 71): |*d*| < 0.2 negligible, 0.2 ≤ |*d*| < 0.5 small, 0.5 ≤ |*d*| < 0.8 medium, and |*d*| ≥ 0.8 large.

To quantify the overlap between the probability distributions of the APG indices in the different clinical groups, the Hellinger distance was calculated for each pairwise comparison, assuming Gaussian distributions. The Hellinger distance ranges from 0 (identical distributions) to 1 (no overlap) and provides a complementary measure to effect size, as it accounts for both differences in central tendency and dispersion (72).

To account for the age difference between groups, an analysis of covariance was performed for each APG index with clinical group as factor and age, together with heart rate, as covariates. The homogeneity-of-regression-slopes assumption was verified through the group × age interaction, and residual normality and homoscedasticity were checked using the Lilliefors and Breusch-Pagan tests. Age-corrected pairwise differences are reported with 95% confidence intervals, and partial eta squared (*η_p_*^2^) is reported as the effect size for the group factor.

## 3 Results

The analysis was conducted on all 133 subjects, stratified into three clinical groups: 45 healthy, 68 hypertensive and 20 diabetic subjects.

### 3.1 Morphological analysis of PPG and APG waveforms

**Figure 2:**
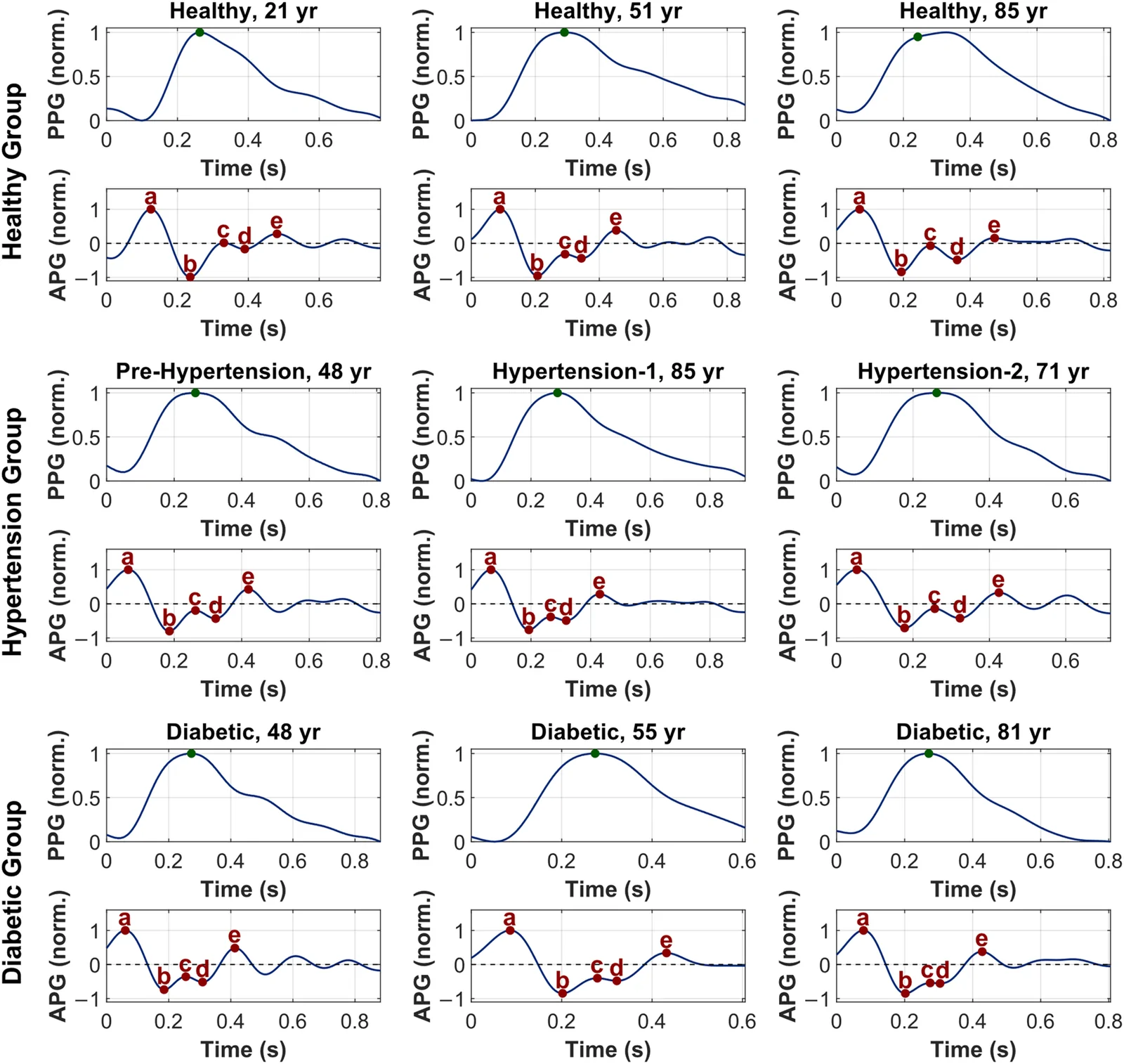
PPG waveforms and corresponding APG waveforms for subjects in the three clinical groups and different age groups.

Figure 2 presents representative examples of normalised PPG and APG waveforms from subjects belonging to the three clinical groups, showing the morphological variations associated with different ages and clinical conditions.

In the healthy group, waveforms show progressive changes with ageing: the 21-year-old subject has a well-defined PPG waveform with a clear dicrotic notch and distinct APG fiducial points, including a prominent d negative wave. With increasing age (51 and 85 years), the dicrotic notch becomes progressively attenuated in the PPG signal, while the APG shows a flattening of the c and d waves, reflecting reduced arterial compliance. In the hypertension group, the pre-hypertensive subject (48 years old) shows a relatively preserved waveform morphology, while subjects with overt hypertension (Stage I, 85 years old; Stage II, 71 years old) show progressively altered APG patterns with decreased c and d waves. In the diabetic group, subjects aged 48 and 55 years present APG morphologies with reduced amplitude of waves c and d, comparable to those observed in older hypertensive subjects.

### 3.2 Correlation with chronological age and distribution across clinical groups

**Figure 3:**
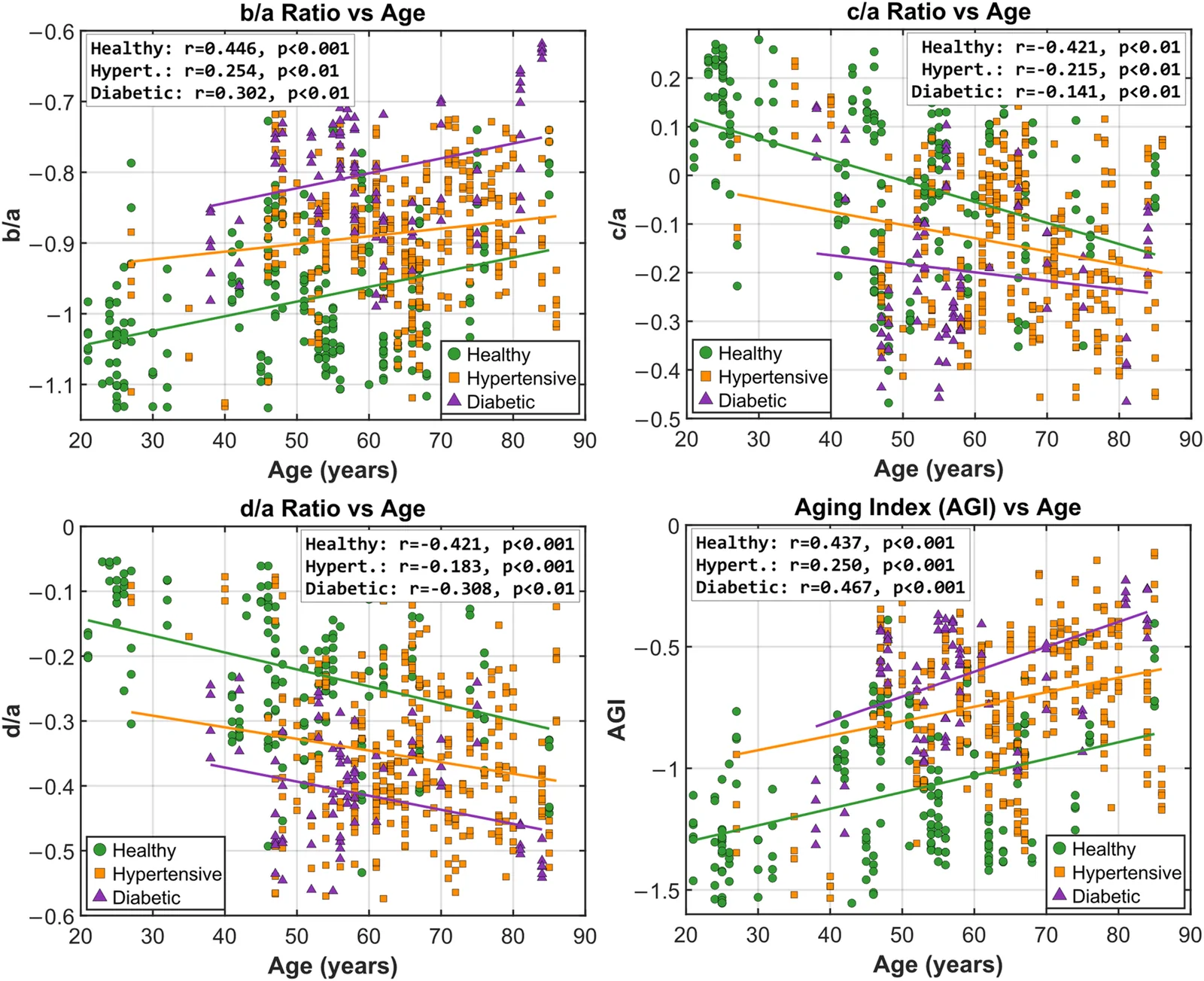
Scatter plots showing the correlation between APG indices (AGI, b/a, c/a and d/a) and chronological age for each clinical group: healthy (green), hypertensive (orange) and diabetic (purple). Linear regression lines are shown for each group with respective Pearson correlation coefficients (*r*) and significance levels (*p*).

Figure 3 shows the relationship between each APG index, calculated for each segment of each subject, and chronological age, stratified by clinical group, with linear regression lines. Pearson correlation coefficients (*r*) and corresponding *p*-values are indicated for each group.

Both the Ageing Index (AGI) and the b/a ratio showed significant positive correlations with age across all three clinical groups. For the AGI, Pearson correlation coefficients were r=0.437 for healthy subjects, r=0.250 for hypertensive subjects, and r=0.467 for diabetic subjects. For the b/a ratio, the corresponding values were r=0.446, r=0.254, and r=0.302, respectively. It is important to note that, at the same chronological age, healthy subjects consistently showed lower AGI and b/a values compared to subjects in the pathological groups; therefore, the regression lines for diabetic and hypertensive subjects are higher than that of healthy subjects.

The c/a and d/a ratios, on the other hand, showed significant negative correlations with age in all groups. For the c/a ratio, the correlation coefficients are: *r* = −0.421 in healthy subjects, *r* = −0.215 in hypertensive subjects and *r* = −0.141 in diabetic subjects. Similarly, significant negative correlations were observed for the d/a ratio: *r* = −0.421 in healthy subjects, *r* = −0.183 in hypertensive subjects, and *r* = −0.308 in diabetic subjects. The healthy group showed the steepest regression slope, indicating a more pronounced age-related decline in d/a values in the absence of cardiovascular disease. Both pathological groups had more negative c/a and d/a values than healthy subjects of the same age, consistent with increased wave reflection and reduced arterial compliance.

**Figure 4:**
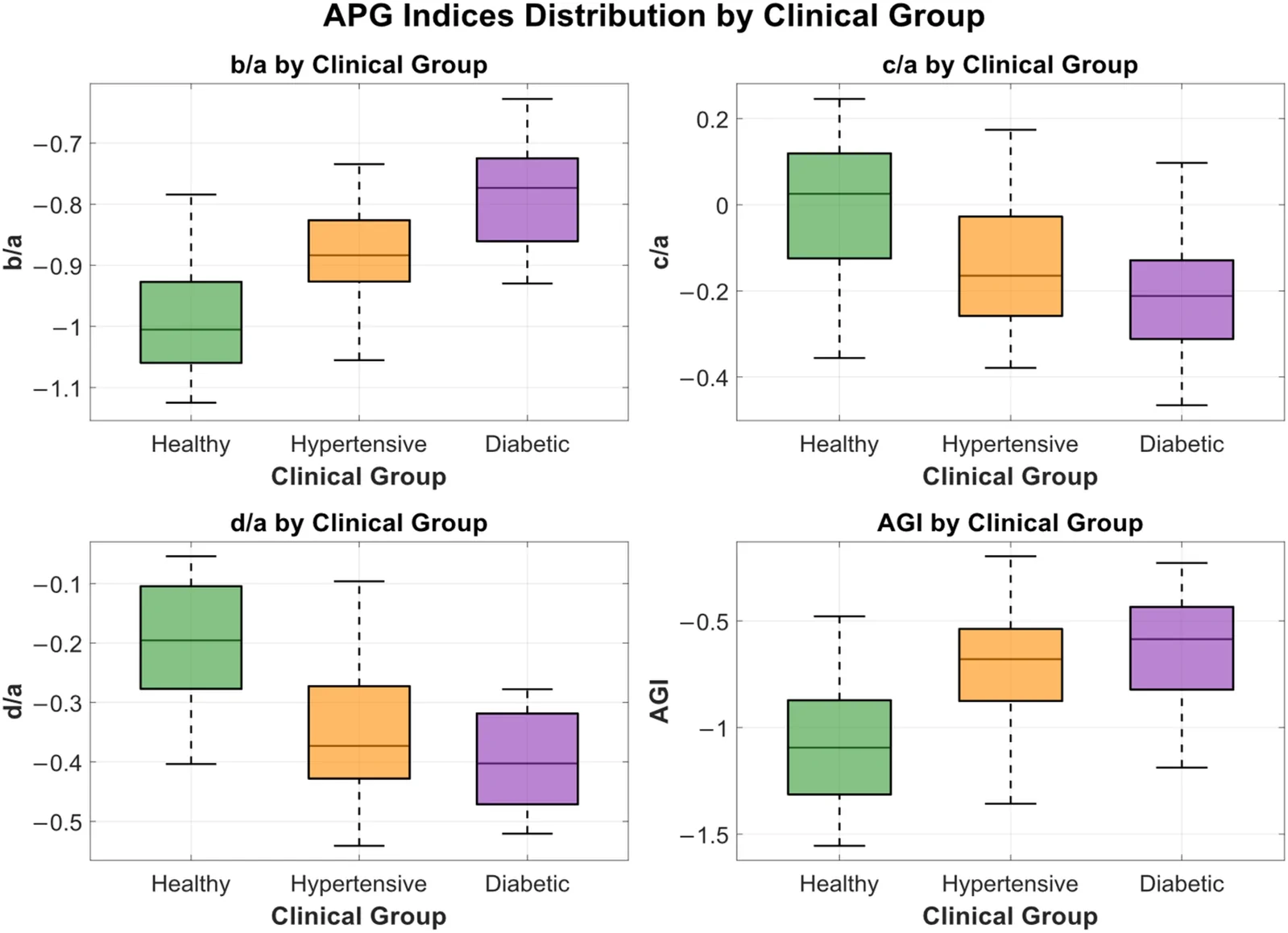
Box plots showing the distribution of APG indices in the three clinical groups.

Table 2 summarises the numerical values of the APG indices for each clinical group, including mean, standard deviation, Pearson's correlation coefficient (*r*) and relative significance value (*p*). These distributions are visualized in Figure 4, which provides box plots for each APG and clinical groups.

**Table 2:** APG index values (mean ± standard deviation), Pearson correlation coefficients (*r*) and significance levels (*p*) for healthy, hypertensive and diabetic subjects.

Clinical group	Statistic	b/a	c/a	d/a	AGI
Healthy subjects	Mean	−0.983	0.001	−0.222	−1.083
	Std. dev.	0.095	0.170	0.108	0.279
	*r*-value (95% CI)	0.446 [0.175, 0.654]	−0.421 [−0.636, −0.145]	−0.421 [−0.636, −0.145]	0.437 [0.165, 0.647]
	*p*-value	<0.001	<0.01	<0.001	<0.001
Hypertensive subjects	Mean	−0.886	−0.142	−0.355	−0.720
	Std. dev.	0.085	0.151	0.110	0.279
	*r*-value (95% CI)	0.254 [0.017, 0.464]	−0.215 [−0.431, 0.025]	−0.183 [−0.404, 0.058]	0.250 [0.012, 0.461]
	*p*-value	<0.01	<0.01	<0.001	<0.001
Diabetic subjects	Mean	−0.792	−0.197	−0.403	−0.647
	Std. dev.	0.094	0.154	0.088	0.270
	*r*-value (95% CI)	0.302 [−0.162, 0.657]	−0.141 [−0.549, 0.322]	−0.308 [−0.660, 0.156]	0.467 [0.031, 0.754]
	*p*-value	<0.01	<0.01	<0.01	<0.001

### 3.3 Verification of statistical assumptions

Parametric test assumptions were verified before group comparisons. The normality of the distribution of each APG index within each group was assessed using the Lilliefors test, and homoscedasticity (homogeneity of variances) was verified using the Levene test. The results are shown in Table 3.

**Table 3:** Verification of ANOVA assumptions for APG indices.

Index	Normality (Lilliefors)			Levene’s Test		Test used
	Healthy (*p*)	Hypert. (*p*)	Diabetic (*p*)	*F*	*p*	
b/a	0.183	0.431	0.500	0.287	0.751	ANOVA
c/a	0.056	0.500	0.500	0.652	0.523	ANOVA
d/a	0.048*	0.017*	0.500	1.057	0.351	Kruskal–Wallis
AGI	0.018*	0.244	0.499	0.393	0.676	Kruskal–Wallis

* *p* < 0.05, normality assumption not satisfied.

For the b/a and c/a indices, data were normally distributed in all three clinical groups (*p* > 0.05 for all), and Levene's test confirmed the homoscedasticity of the variances (b/a: *F* = 0.287, *p* = 0.751; c/a: *F* = 0.652, *p* = 0.523). One-way ANOVA was therefore used for these two parameters.

For the d/a and AGI indices, normality was not satisfied in at least one group (d/a: Healthy *p* = 0.048, Hypertensive *p* = 0.017; AGI: Healthy *p* = 0.018), despite homoscedasticity being verified (d/a: *F* = 1.057, *p* = 0.351; AGI: *F* = 0.393, *p* = 0.676). For these two parameters, the Kruskal–Wallis test was therefore used as a non-parametric alternative.

### 3.4 Omnibus test and pairwise comparisons

The omnibus test revealed statistically significant differences between the three clinical groups for all four APG indices (Table 4). Since all omnibus differences were significant (*p* < 0.001), pairwise *post-hoc* comparisons were performed to identify which groups differed from each other.

**Table 4:** Omnibus test results for APG indices across clinical groups.

Index	Test	Statistic	df	*p*-value
b/a	ANOVA	*F* = 43.97	2, 130	<0.001
c/a	ANOVA	*F* = 15.74	2, 130	<0.001
d/a	Kruskal–Wallis	*χ*^2^ = 44.38	2	<0.001
AGI	Kruskal–Wallis	*χ*^2^ = 37.28	2	<0.001

For b/a and c/a (ANOVA), Tukey's HSD test was used. For d/a and AGI (Kruskal–Wallis), Dunn's test with Bonferroni correction was used. The results of the *post-hoc* comparisons are shown in Table 5.

**Table 5:** Pairwise comparisons between clinical groups: *post-hoc p*-values, effect size (Cohen's *d*) and distribution overlap (Hellinger distance) for all APG indices.

Index	Comparison	*Post-hoc p*	Cohen's *d*	95% CI	Hellinger	Effect
b/a	Healthy vs. hypert.	<0.001	−1.26	[−1.68, −0.84]	0.42	Large
	Healthy vs. diabetic	<0.001	−2.35	[−3.02, −1.67]	0.71	Large
	Hypert. vs. diabetic	<0.001	−1.19	[−1.73, −0.66]	0.40	Large
c/a	Healthy vs. hypert.	<0.001	0.92	[0.52, 1.32]	0.32	Large
	Healthy vs. diabetic	<0.001	1.22	[0.64, 1.80]	0.42	Large
	Hypert. vs. diabetic	0.281	0.42	[−0.10, 0.93]	0.15	Small
d/a	Healthy vs. hypert.	<0.001	1.44	[1.00, 1.88]	0.48	Large
	Healthy vs. diabetic	<0.001	2.10	[1.44, 2.76]	0.67	Large
	Hypert. vs. diabetic	0.361	0.49	[−0.02, 0.99]	0.22	Small
AGI	Healthy vs. hypert.	<0.001	−1.33	[−1.76, −0.90]	0.45	Large
	Healthy vs. diabetic	<0.001	−1.57	[−2.17, −0.96]	0.52	Large
	Hypert. vs. diabetic	1.000	−0.26	[−0.76, 0.24]	0.09	Small

The comparison between healthy and hypertensive subjects was statistically significant for all four APG indices (*p* < 0.001 for all). Similarly, the comparison between healthy and diabetic subjects was significant for all parameters (*p* < 0.001 for all). The comparison between hypertensive and diabetic subjects was statistically significant only for the b/a index (*p* < 0.001), while it did not reach statistical significance for c/a (*p* = 0.281), d/a (*p* = 0.361) and AGI (*p* = 1.000). To quantify the practical extent of the differences between the groups, Cohen's d was calculated as a measure of effect size.

**Figure 5:**
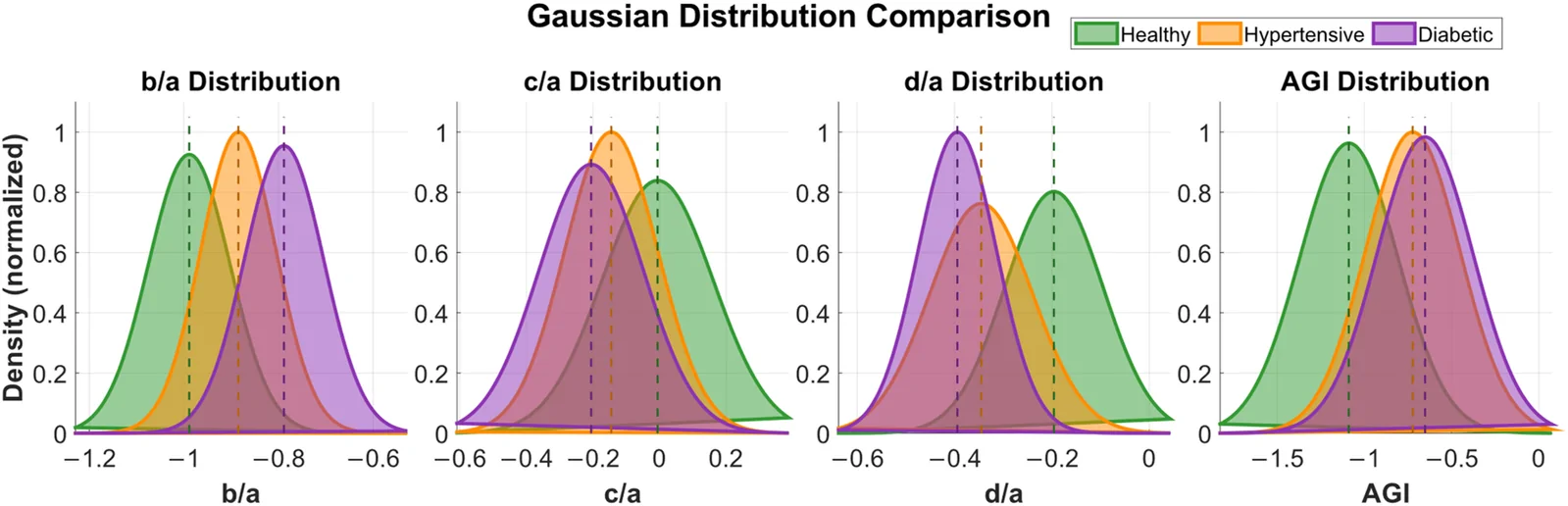
Normalised Gaussian distributions of APG indices for the three clinical groups. The vertical dotted lines indicate the mean value for each group.

The normalised Gaussian curves, fitted to the data of the three clinical groups, are shown in Figure 5.

**Figure 6:**
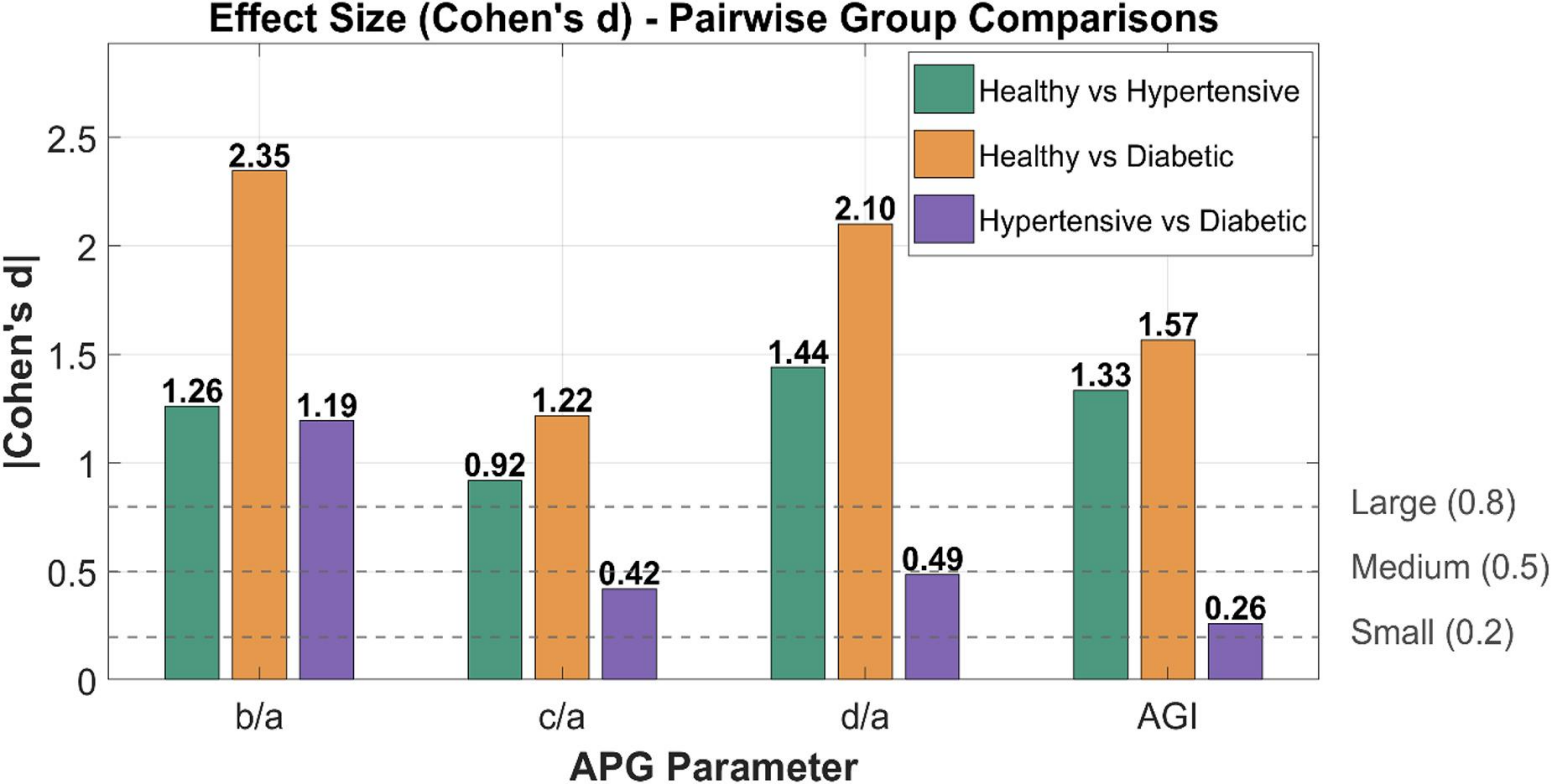
Comparison of effect sizes (Cohen's *d*) between APG parameters and comparisons between clinical groups.

This graphical representation allows for an immediate visual comparison of the central tendency and dispersion among the populations examined. To assess the degree of overlap between the distributions of APG parameters in the different clinical groups, Hellinger's distance was calculated, assuming Gaussian distributions. Figure 6 shows Cohen's *d* values for pairwise comparisons between clinical groups for all APG parameters.

The comparison between healthy subjects and hypertensive subjects showed large effects (|*d*| > 0.8) for all indices: b/a (|*d*| = 1.26), c/a (|*d*| = 0.92), d/a (|*d*| = 1.44) and AGI (|*d*| = 1.33). The Hellinger distance for this comparison ranged from 0.32 (c/a) to 0.48 (d/a), indicating a high to moderate overlap of distributions.

The comparison between healthy subjects and diabetic subjects produced the highest effect sizes for all parameters: b/a (|*d*| = 2.35), c/a (|*d*| = 1.22), d/a (|*d*| = 2.10) and AGI (|*d*| = 1.57), all well above the conventional threshold for large effects (|*d*| > 0.8). Hellinger's distance ranged from 0.42 (c/a) to 0.71 (b/a), the latter corresponding to a low overlap between distributions.

The comparison between hypertensive and diabetic subjects showed lower effect sizes: c/a (|*d*| = 0.42), d/a (|*d*| = 0.49) and AGI (|*d*| = 0.26), all classifiable as small. The Hellinger distance for these three indices was very low (c/a: 0.15; d/a: 0.22; AGI: 0.09), indicating an almost complete overlap of the distributions. For the b/a index, on the other hand, the effect size remained large (|*d*| = 1.19) with a Hellinger distance of 0.40, and the *post-hoc* comparison was statistically significant (*p* < 0.001).

Prior to interpreting the analysis of covariance results, the underlying model assumptions were evaluated. The homogeneity-of-regression-slopes assumption was satisfied for all indices (group × age interaction, *p* = 0.60–0.94), and residual normality and homoscedasticity were confirmed. With age and heart rate taken into account, clinical group remained a significant determinant of all four indices (b/a: *F* = 32.8, *p* < 0.001, *η_p_*^2^ = 0.35; c/a: *F* = 7.98, *p* < 0.001, *η_p_*^2^ = 0.12; d/a: *F* = 24.2, *p* < 0.001, *η_p_*^2^ = 0.29; AGI: *F* = 15.2, *p* < 0.001, *η_p_*^2^ = 0.20). The age-corrected pairwise differences between groups confirmed significant differences between healthy and both pathological groups for all indices, and between hypertensive and diabetic participants for b/a (difference 0.105, 95% CI 0.066–0.144, *p* < 0.001), as well as for c/a and d/a. Age remained a significant covariate for all indices (*p* < 0.001), whereas heart rate contributed independently to d/a (*p* = 0.003) and c/a (*p* = 0.047) without altering the group effects.

## 4 Discussion

Our findings indicate that APG indices derived from wearable-compatible PPG signals differ across cardiometabolic health states and reflect vascular ageing in healthy, hypertensive and diabetic participants. Table 6 summarises the APG indices analyzed in this work (b/a, c/a, d/a and AGI), outlining their mathematical formulations and their physiological and clinical interpretations as described in prior studies. The results are consistent with the proposed role of APG indices as candidate markers of cardiometabolic vascular status (73) and reveal distinct patterns for each group (74). To the best of the authors' knowledge, there are no studies in the literature that simultaneously correlate APG indices with multiple heterogeneous clinical groups (healthy, hypertensive, and diabetic subjects) considering a wide age range.

**Table 6:** APG indices: formulas and clinical interpretation.

Index	Formula	Clinical interpretation
b/a ratio	b ÷ a	Increases with age. Indicator of increased arterial stiffness and reduced distensibility of peripheral arteries. Marker of atherosclerosis (28, 73)
c/a ratio	c ÷ a	Decreases with age. Reflects the late systolic component and vascular compliance (45)
d/a ratio	d ÷ a	Decreases with age. Reflects the increase in late systolic pressure in the ascending aorta caused by peripheral reflected waves. Predictor of cardiovascular mortality (75, 76)
AGI	(b − c − d − e) ÷ a	The most comprehensive index for assessing vascular ageing. Increases with age. Useful for screening for atherosclerosis and cardiovascular risk stratification (77)

In our study population of 133 subjects, we observed that the Ageing Index (*r* = 0.250–0.467, *p* < 0.001) and the b/a index (*r* = 0.254–0.446, *p* < 0.001) showed significant positive correlations with age, while the c/a index (*r* = −0.141 to −0.421, *p* < 0.01) and d/a index (*r* = −0.183 to −0.421, *p* < 0.001) showed significant negative correlations. This bidirectional pattern reflects the complex haemodynamic changes associated with vascular ageing and confirms the theoretical framework proposed in the original literature on APG: the b/a ratio and ageing index are inversely correlated with arterial distensibility and tend to increase with age and the presence of cardiovascular risk factors, reflecting greater arterial wall stiffness, while the c/a and d/a ratios decrease progressively as the vascular wall becomes stiffer (78).

The morphological changes described in Section 3.1 and illustrated in Figure 2 follow the pattern expected from progressive arterial stiffening: attenuation of the dicrotic notch (79) and flattening of the c and d waves with advancing age in healthy participants. A comparable but more marked pattern was observed in participants with established hypertension, reflecting the impact of chronic pressure overload on the arterial wall (80). Notably, the diabetic group showed an acceleration of vascular alterations, evident even in relatively young subjects who presented APG morphologies typically associated with advanced vascular aging, characterized by a reduced amplitude of the c and d waves, comparable to that observed in much older hypertensive subjects (81, 82). These morphological observations suggest that diabetes mellitus can induce premature vascular changes (83, 84) that are qualitatively similar to, but temporally accelerated compared to, those caused by ageing and hypertension alone (85). A key advantage of the APG is that these progressively attenuating features, including the dicrotic notch, which becomes increasingly difficult to discern on the raw PPG with age and disease, remain identifiable and measurable on the second derivative, allowing them to be characterised quantitatively rather than noted only as the loss of a visual landmark.

The results obtained in this study (Table 2) are consistent with previous studies in the literature, including the seminal work of Takazawa et al. (45), who were the first to demonstrate the clinical utility of the APG signal for assessing vascular ageing and arterial stiffness. In Takazawa's study, conducted on 600 subjects (50 men and 50 women for each decade from the third to the eighth), strong correlations were reported between APG indices and age, with correlation coefficients of *r* = 0.75 for the b/a ratio, *r* = −0.67 for c/a, *r* = −0.72 for d/a, and *r* = 0.80 for the AGI index. The weaker correlations observed here likely reflect two differences in design: Takazawa's cohort excluded participants with documented cardiovascular disease, whereas ours was deliberately stratified by clinical condition, and our group sizes did not permit the systematic decade-by-decade sampling adopted in that study. Age is therefore a major determinant of APG indices in both healthy and diseased individuals, consistent with the progressive loss of elastin and accumulation of collagen in the arterial wall (86). Inoue et al. (75), in a longitudinal study of 4,373 Japanese women aged 50–79 years with a median follow-up of 9 years, demonstrated that the d/a ratio was significantly and inversely correlated with age (*r* = −0.172, *p* < 0.001). More recently, another study (76) investigated the relationship between the d/a ratio and vascular stiffness through both *in vitro* and *in vivo* approaches. The *in vivo* analysis on healthy volunteers aged 26–63 years revealed a strong negative correlation between the d/a ratio and arterial stiffness (*R* = −0.90), further supporting the role of this parameter as a non-invasive index of vascular ageing.

All four APG indices differed significantly across the three clinical groups (Table 4), with consistently large effect sizes in the comparisons between healthy and pathological participants (Table 5), indicating that APG parameters are sensitive to the vascular alterations associated with cardiovascular disease. Among these, the d/a ratio (|*d*| = 1.44) showed the largest effect size in the healthy-vs.-hypertensive comparison, consistent with the established role of this index as a marker of peripheral wave reflection and arterial stiffness. The corresponding Hellinger distances, however, indicate that despite the large effect sizes the distributions of individual APG indices in healthy and hypertensive subjects still overlap considerably, suggesting that a single APG parameter may not be sufficient for reliable classification at the individual level. This reinforces the potential advantage of combining multiple APG indices, or integrating them with additional clinical variables, to improve discriminative performance, which would need to be established in dedicated diagnostic-accuracy studies.

These findings are supported by Liang et al. (87), who analysed 125 features extracted from the PPG and its derivatives in relation to systolic blood pressure and reported a strong correlation between the composite index (b-c-d)/a and systolic blood pressure (*r* = 0.69, *p* < 0.001), and by Yao and Liu (88), who identified the b/a and d/a ratios as unique markers of hypertension through ANOVA-based feature selection. Notably, the prominent role of d/a observed in our data aligns with the prospective study by Otsuka et al. (89), who demonstrated that the d/a ratio independently predicted the future development of hypertension in 902 normotensive middle-aged men, suggesting that stiffening of small and medium-sized muscular arteries may precede the onset of elevated blood pressure. Taken together, these results support the potential role of APG indices, and in particular the d/a index, as biomarkers for hypertensive risk stratification in preventive cardiovascular medicine.

The healthy-vs.-diabetic comparison yielded the largest effect sizes across all four indices (Table 5), with the b/a ratio (|*d*| = 2.35) showing the smallest distributional overlap of any comparison performed in this study. These results suggest that diabetes is associated with a particularly pronounced alteration of APG-detectable vascular properties, likely reflecting the combined effect of metabolic dysfunction and accelerated arterial stiffening. The direction and magnitude of the b/a difference are consistent with the progressive arterial stiffening expected in the presence of chronic metabolic disease.

These findings are consistent with those of Kawada and Otsuka (90), who reported a significant association between the d/a ratio and metabolic syndrome risk (OR = 0.10, 95% CI: 0.01–0.73, *p* < 0.05), while noting that APG indices in their factor analysis clustered primarily with age-related vascular parameters rather than with metabolic components. This may support the hypothesis that APG-detectable arterial stiffening reflects the vascular consequences of metabolic disease and may contribute to its progression (91, 92). The potential of the b/a ratio for monitoring glycaemic control was further supported by Usman et al. (93), who reported significant differences in b/a values across HbA1c levels in subjects with type 2 diabetes. Moreover, Tayade et al. (94) demonstrated that the b/a ratio correlated significantly with the ankle-brachial index and increased progressively with the severity of peripheral arterial disease, a frequent complication of diabetes (95), with an age-related pattern (*r* = 0.38, *p* = 0.036) consistent with our observations.

These results and comparisons with literature support the ability of APG parameters to identify vascular alterations associated with cardiovascular disease. Interestingly, the comparison between hypertensive and diabetic subjects revealed lower effect sizes for c/a, d/a, and AGI, with Hellinger distances indicating almost complete distributional overlap (0.09–0.22). This suggests that these indices may be less effective in discriminating between different cardiovascular conditions once vascular damage has occurred, as both hypertension and diabetes converge towards similar patterns of arterial stiffening. However, the b/a ratio maintained a large effect size even in this comparison, potentially indicating its greater responsiveness to the specific pathophysiological mechanisms underlying diabetic vasculopathy, particularly the structural changes in the arterial wall induced by chronic hyperglycaemia, which may differ from the predominantly haemodynamic alterations caused by hypertension.

## 5 Limitations

This study has some limitations that should be acknowledged. First, the size of the diabetic group (*n* = 20) was relatively small compared to the healthy (*n* = 45) and hypertensive (*n* = 68) groups, which could limit the statistical power of pairwise comparisons involving diabetic subjects and the generalizability of the results to the wider diabetic population. In addition, most diabetic subjects also presented some degree of elevated blood pressure, so the diabetic group represents diabetes with frequently concomitant hypertension rather than isolated diabetes. This overlap makes it difficult to isolate the independent effect of diabetes mellitus on APG indices, and the direct comparison between the hypertensive and diabetic groups should be interpreted in this light. The particularly pronounced alterations observed in the diabetic group may therefore reflect the combined effect of the hemodynamic and metabolic burden of both conditions, rather than the effect of diabetes alone. A further limitation concerns the clinical information available in the public dataset. Variables such as HbA1c levels, treatment history, and duration of hypertension or diabetes were not available and could not be assessed, although they may contribute to variability in APG indices and influence the likelihood of arteriosclerotic changes. Future prospective studies incorporating comprehensive clinical characterization and longitudinal follow-up will be valuable for elucidating their influence on APG-derived parameters. Finally, although only high-quality segments were selected, the segments available in the database were short in duration and contained a limited number of analysable cardiac cycles for each subject. Longer recordings, lasting several minutes, would allow a greater number of waveforms to be obtained, improving the robustness of the fiducial point estimation and the stability of the derived APG indices, while reducing intra-subject variability.

## 6 Conclusion

This study shows that indices derived from the APG are significantly associated with chronological age and differ substantially between healthy, hypertensive and diabetic individuals, supporting their potential as non-invasive digital biomarkers for monitoring cardiometabolic health. All APG indices analysed (b/a, c/a, d/a and AGI) showed large effect sizes in differentiating healthy subjects from pathological groups, indicating their clinical usefulness in identifying vascular alterations associated with arterial hypertension and diabetes. In particular, the b/a ratio showed the largest effect sizes not only in detecting the presence of cardiovascular disease, but also in discriminating between different pathological conditions, suggesting its potential specificity for the pathophysiological mechanisms of diabetic vasculopathy.

From a translational perspective, these findings support the potential integration of APG-based analysis into wearable devices for continuous, non-invasive cardiovascular monitoring. Given that PPG sensors are already widely embedded in consumer electronics such as smartwatches and smart rings, the real-time computation of APG indices could, subject to further clinical validation, support cardiovascular risk screening in primary care, remote patient monitoring, and population-level health surveillance, contributing to the shift towards preventive and personalised cardiovascular medicine.

Future studies should extend the present findings by evaluating the diagnostic and prognostic performance of APG indices in larger, independent cohorts with longitudinal follow-up and adjudicated cardiovascular outcomes. Such validation would be essential to establish whether the between-group differences observed here translate into clinically actionable screening tools. In parallel, the integration of machine learning and deep learning algorithms could improve the accuracy of cardiovascular condition classification, enabling the development of more robust predictive models and accelerating the clinical translation of APG-based digital biomarkers into large-scale cardiovascular monitoring applications.

## Data Availability

The PPG-BP dataset analysed in this study is publicly available on Figshare at https://doi.org/10.6084/m9.figshare.5459299 (Liang Y, Liu G, Chen Z, Elgendi M. PPG-BP Database). The dataset is described in the accompanying data descriptor: Liang Y, et al. A new, short-recorded photoplethysmogram dataset for blood pressure monitoring in China. Sci Data. (2018) 5:180020.
